# EP03 Parechovirus in a pathologist

**DOI:** 10.1093/rap/rkaa052.002

**Published:** 2020-11-03

**Authors:** Claire Masih, Michael Dologhan, Andrew Cairns

**Affiliations:** Musgrave Park Hospital, Belfast, United Kingdom

## Abstract

**Case report - Introduction:**

A 42-year-old pathologist presented with a 1-week history of muscle pain and subjective weakness. CK level on 2 occasions was >3000.

The patient was systemically well with no past medical history, medication, or foreign travel. He had 1-day history of shivering with no recorded pyrexia. He reported pain in his proximal muscles and neck and subjective muscle weakness and lack of finger dexterity with no objective findings.

**Case report - Case description:**

Autoantibody panel and inflammatory markers were performed which were normal. Full blood count with differential white cell count including eosinophils was normal. There was a modest rise in transaminases. Myositis panel was negative. Full viral screen was positive for parechovirus with titre of 30 on several samples. MRI proximal musculature showed increased fluid signal in the perifascial region of both thighs primarily involving the hamstrings, not definitive for myositis but suggestive of fasciitis.

**Case report - Discussion:**

Parechovirus is a picornavirus, often causing mild gastrointestinal or respiratory illness but has been associated with epidemic myalgia and myositis during outbreaks of parechovirus in a Japanese population.

The patient improved spontaneously with CK reduced to 187 and improved symptoms after 1 week. We expect a good outcome and will review on patient's request if necessary.

**Case report - Key learning points:**

Parechovirus can cause myofasciitis which is usually mild and self-limiting. It can be associated with elevated CK, transaminases and MRI findings and can be confirmed on respiratory viral swab.

